# The Mode of Communication as a Driver of Sustainable and Equitable Asymmetric Common Pool Resource Use

**DOI:** 10.1007/s00267-023-01825-w

**Published:** 2023-04-28

**Authors:** Kaisa Herne, Jonathan Kuyper, Olli Lappalainen

**Affiliations:** 1 Tampere University, Faculty of Management and Business, Politics, Tampere, Finland; 2 University of Oslo, Department of Political Science, Oslo, Norway; 3 University of Turku, Economics, Turku, Finland

**Keywords:** Asymmetric Common Pool Resource, Communication, Deliberation, Power Imbalance, Experiment

## Abstract

Most experimental studies on common pool resource usage focus on situations in which actors are in symmetric positions when they use the resource. Many real-world cases do not fit this scenario because users are in asymmetric positions regarding their ability to benefit from the resource. Examples range from irrigation systems to climate change mitigation. Moreover, while there is large evidence on the effects of communication on social dilemmas, few studies focus on different modes of communication. We compare the effects of unstructured and structured communication on the provision of an infrastructure for a common pool resource and appropriation of the provided resource. Structured communication applied rules that are based on the ideals of democratic deliberation. Participants made contribution and appropriation decisions in an incentivized experiment. In the experiment, both communication and deliberation increased contributions in comparison to a baseline. Interestingly, deliberation attenuated the effect of the player position more than communication. Our results suggest that deliberation may be useful for overcoming asymmetric commons dilemmas in the field.

## Introduction

Climate change and biodiversity loss are sad examples of resource overuse at the global scale. They are also examples of the tragedy of the commons, that is, a consistent overuse of a common pool resource leading to sub-optimal group-level outcomes (Hardin 1968). Despite these worrying examples, much work has also demonstrated success: especially local and small-scale communities can often self-govern sustainable common pool resource usage (Ostrom 1990; Ostrom 2011). While there are many potential mechanisms underlying this cooperation, previous research has established that communication is crucial (Ostrom 2006; Ostrom 2009).

While many common pool resources, such as fisheries or forests, appear through natural processes, others require human action to make the resource available for appropriation. We can therefore separate natural and man-made common pool resources (Ostrom and Gardner 1993). Irrigation systems typically require a construction of an infrastructure through which the appropriation of water is possible. There is therefore a two-stage collective action problem including the provision and appropriation stages (Cardenas et al. 2010). We study such problems, that is, users first decide how much to *contribute* to creating an infrastructure through which a resource is made available, after which they decide how much to *extract* from the resource (Cardenas et al. 2010, Janssen et al. 2011a, 2011b, Janssen et al. 2015). In our case, contribution decisions determine how much of the resource is available for use. The so provided common pool resource is rival but non-excludable indicating that one’s extractions from the resource reduce others’ possibilities to extract from the resource. For simplicity, we talk about contributions to and extractions from a common pool resource, although the contribution stage is not rival.

Sustainable common pool resource use appears especially tricky when extractions take place sequentially (Cardenas et al. 2010). A paradigm example of such an *asymmetric common pool resource* is an irrigation system, where head-enders have priority over the use of the resource over tail-enders. Asymmetric resources are problematic because users hold *different positions of power* regarding their ability to benefit from the resource. It is also noteworthy that participants’ knowledge of sequential extractions can influence their willingness to contribute to the provision of the infrastructure (Cardenas et al. 2010). Existing evidence from a field study shows that communication can improve sustainable use under asymmetric conditions (Cardenas et al. 2013, Janssen et al. 2011b), but also that the effect of communication can vary depending on the context (Cardenas et al. 2010).

We ask whether the *mode of communication* matters. Exiting literature does not say much about different forms of communication, although they are relevant for organizing communication in the field. We compare the effects of structured and unstructured modes of communication on sustainable and equitable common pool resource use. Resource use is sustainable if users can maintain the infrastructure for providing the resource over a reasonable long period of time, and it is equitable if unearned asymmetries in one’s ability to use the resource are not reflected in the provision of the infrastructure or in the use of the common pool resource. In our study, structured communication is inspired by the theory of *deliberative democracy* (Elster 1998; Dryzek 2000; O’Flynn 2022). Deliberative democracy provides a normative model of democracy in which reason-giving among free and equal citizens is perceived necessary for legitimate democratic decisions. During recent decades, deliberative democracy has inspired a wide range of empirical research (Gastil and Dillard 1999; Luskin et al. 2002; Fishkin and Luskin 2005; Grönlund et al. 2015; Gerber et al. 2018; Farrell et al. 2020; Knobloch et al. 2020). The reason for turning to deliberative democracy when creating structured communication is its alleged potential for tackling environmental issues and promoting sustainable preferences (Goodin 1992; Smith 2003; Grönlund et al. 2022; Willis et al. 2022), as well as for neutralizing power imbalances (Cohen and Rogers 2003).

To examine the effects of the modes of communication, we carried out a laboratory experiment on an asymmetric common pool resource. In the baseline, communication was not allowed, whereas the two treatment conditions allowed either unstructured or structured communication. To structure communication we applied rules of discussion that emphasized justifications, perspective-taking and respect of other participants. Parallel rules are typically applied in empirical applications of democratic deliberation (Grönlund et al. 2014). While we did not observe a statistically significant difference between unstructured and structured communication regarding total contributions, we found that structured communication attenuated the player position effect more than unstructured communication.

We will next outline the relevant literature and formulate hypotheses, after which we introduce the experimental design and procedures. We then report results in terms of contributions to the common pool resource infrastructure and extractions from the common pool resources, and a short analysis of chat entries. The final section concludes and discusses the relevance of our results.

## Sustainable and Equitable use of Asymmetric Common Pool Resources

The key insight of work on common pool resources – and public goods more broadly – is that overuse and free-riding do not always emerge (Ostrom 1990; Anderies et al. 2011). Studies of common pool resources show that localized rules help individuals take ownership over governance systems and solve common pool resource problems (Ostrom et al. 1999). Likewise, the ability to monitor one-another, especially in the absence of high levels of social trust, is an important determinant of sustainable common pool resource usage (Gibson et al. 2005). While most evidence on common pool resources concern symmetric resources, there is also evidence that contributions are made in making asymmetric resources available (Cardenas et al. 2010; Janssen et al. 2011a, b; Janssen et al. 2015).

Laboratory experiments have further honed-in on’micro-situational variables’ which account for common pool resource use. The most robust observations concern communication and group size (Ostrom 2006; Hackett et al. 1994). Evidence show that communication has a robust and positive relationship with sustainable common pool resource use (Meinzen-Dick et al. 2018). Regarding group size, contributions to a public good tend to go up with group size, whereas this is not the case in common pool resource games, perhaps due to scarcity (Isaac and Walker 1988). There is rather robust evidence that the possibility to punish free riders increases contributions to public goods (Fehr and Gächter 2000; Fehr and Rockenbach 2003; Choi and Ahn 2013; Lierl 2016). Regarding common pool resources, punishment tends to enhance sustainable resource use especially when connected to communication (Ostrom et al. 1992, Janssen et al. 2010).

Why is communication so important for solving common pool resource problems? Communication is called “cheap talk” if it is costless and nonbinding (Duffy and Feltovich 2002). Such talk can increase contributions to public goods (Palfrey and Rosenthal 1991; Oprea et al. 2014; Palfrey et al. 2017). Communication seems to influence contributions by several mechanisms: by increasing understanding of the choice situation; by helping participants to coordinate their beliefs and actions; by changing expectations about others’ behavior; by generating norms of cooperation; and by generating group identity and solidarity (Balliet 2010; Janssen et al. 2010; Lopez and Villamayor-Tomas 2017; Koessler et al. 2021). Communication seems relevant for building trust (Ben-Ner and Putterman 2009), which in turn promotes cooperation (Chaudhuri et al. 2002; Gächter et al. 2004). Yet evidence on the effects of communication in the case of asymmetric common pool resources is scarce. Cardenas et al. (2010) compare conditions with and without communication in a field study, whereas Janssen et al. (2011a, b) report on laboratory experiments where communication is allowed in all treatment conditions. Based on large evidence from social dilemma situations in general, we hypothesize that giving participants *an opportunity to communicate increases contributions to the asymmetric common pool resource infrastructure (H1)*.

Despite robust evidence on the effects of communication, common pool resource overuse is observed in the field although communication is seldom prevented in real-world interactions. This suggests that communication may sometimes be difficult even though not actively prevented. Yet it is also possible that sometimes communication as such is not sufficient. We thereby investigate the possibility that different modes of communication might be relevant for solving common pool resource problems. Existing research does not say much about which types of communication work best in promoting cooperation. Kossler et al. (2021) show that structuring communication with information about certain aspects of a social dilemma enhanced cooperation. We turn to the ideals of deliberative democracy to structure communication.

Deliberative discussion can influence behaviour by several mechanisms.^1^ Asking participants to justify their claims can influence behaviour because acting fairly is easier to justify (de Kwaadstenie et al. 2007, De Cremer and van Dijk, 2009), and because it can promote norm-abiding behaviour (De Kwaadsteniet et al., (2007); Xiao 2017). Prompting perspective taking tends to decrease self-interested behaviour (Batson et al. 1997) as well as to promote pro-social and pro-environmental behaviour (Heinz and Koessler (2021); Ortiz-Riomalo et al. 2021). It is noteworthy though that under some conditions, prompting perspective-taking may in fact increase self-interested behaviour (Epley et al. 2006; Wald et al. 2017). We assume that the combination of justifications, perspective-taking and respect for others create a mind-set characterized by trust in others’ cooperation (cf. Gollwitzer and Keller 2016). We hypothesize that engaging in *structured communication increases contributions to the asymmetric common pool resource infrastructure more than unstructured communication (H2)*.

Many common pool resources have structures where spatial or temporal configurations systematically benefit some actors over overs. Experimental research shows that asymmetric common pool resources induce a *position effect*: those who come first in the sequential order tend to make bigger requests and use more of the resource than those who are positioned later in the order (Rapoport et al. 1993: Budescu et al. 1995: Budescu and Au 2002). A related observation is that tail-enders seek to punish head-enders for unfair behaviour by withholding contributions and thus stymying the collective usage of the resource (Janssen et al. 2011a). This observation seems to hold both in the laboratory and in the field (Janssen et al. 2011b). Communication can dampen this negative cycle, increase cooperation, and solidify the sustainable usage of common pool resources (Janssen et al. 2011a). In a sequential dictator game with a similar asymmetric structure, communication attenuated the position effect (Wolf and Dron 2020).

Contributions to the common pool resource infrastructure can reflect players’ expectations about their ability to use the resource. If later movers anticipate that first movers will extract more than an equal share of the resource, later movers have less incentives to contribute. Moreover, later movers can react to their experiences from already played rounds by withdrawing contributions if nothing, or very little, is left to them in previous rounds. We therefore hypothesize that *without communication, contributions to the asymmetric common pool resource infrastructure decrease as a function of player position (H3)*.

From the point of promoting sustainable use of asymmetric common pool resources, the potential of deliberation to attenuate power imbalances is especially interesting (Cohen and Rogers 2003). In deliberation, the argument goes, all participants are given equal voice and they are required to respect others, as well as put forth justification for presented arguments. For these reasons, the force of the better argument (Habermas 1996), rather than power relationships per se, should be reflected in the outcomes of deliberation. Others have, however, raised doubts about the ability of deliberation to neutralize power (Bagg 2018), and direct empirical evidence on the matter is largely missing. There is still evidence that deliberation increases several civic skills such as trust and perspective-taking (Grönlund et al. 2010; Grönlund et al. 2017; Muradova 2020). If people tend to see things from others perspective and if they trust in others’ cooperation, free-riding is likely to decrease. We assume that structured communication according to rules for deliberation is more effective than unstructured communication in building trust in others’ cooperation. We therefore hypothesize that *structured communication attenuates the position effect on contributions to the asymmetric common pool resource infrastructure more than unstructured communication (H4)*.

Finally, the possibility to extract from the resource depends on contributions to the infrastructure for providing the resource. If structured communication endorses most contributions, it also allows most to extract. We assume that structured communication yields an equal distribution of extractions because deliberative rules prompt justifications and because acting fairly is easier to justify to others. Moreover, the rules promote perspective-taking and respect, both of which are also likely to endorse fairness. For these reasons, we hypothesize that *structured communication leads to a more equal distribution of extractions from the asymmetric common pool resource compared to unstructured communication or no communication (H5)*.

## Experimental Design and Procedures

We study a social dilemma situation in which players make simultaneous decisions about their contributions to a common pool resource infrastructure but where extractions from the resource are done sequentially (Janssen et al. 2010). Following Janssen et al. (2011a, b), we use a payoff function with a threshold sum of contributions that must be reached before the resource produces net positive returns. If enough is contributed, participants have an opportunity to earn considerably more than their initial endowment.

We assigned participants into three experimental conditions. In *Baseline*, the asymmetric common pool resource game was played without possibilities to communicate. In *Communication*, participants had an opportunity to use a chat box to communicate with their group members before each contribution and extraction decision. In *Deliberation*, participants were given communication rules before using the chat. The rules promoted justifications, perspective-taking, and respect, which was specified as a denial of using threats or abusive expressions. These rules approximate the central ideals of deliberation, and they are similar to discussion rules commonly applied in deliberative mini-publics (Grönlund et al. 2021).^2^ It is yet noteworthy that chat discussions were a loose approximation of the ideals of democratic deliberation: discussions lasted for one minute per round, they involved only three participants, discussions were not moderated, and obeying the rules was not monitored. Moreover, the topic of discussion – how to contribute to and extract from an experimental common pool resource – does not compare to complex political issues where participants’ values, ideologies and identities are relevant. Rather than engaging in genuine democratic deliberation, participants were treated with priming them with a *deliberative mindset* (cf. Gollwitzer and Keller 2016). The treatment is somewhat similar with priming participants with perspective-taking towards stigmatized groups (Batson et al. 1997).

We conducted a computerized laboratory experiment (z-tree, Fischbacher 2007) to study the influence of communication and deliberation on the use of an asymmetric common pool resource. Participants to the experiment were recruited via an online system from a pool of registered subjects (ORSEE, Greiner 2015). Upon arrival in the laboratory, participants were assigned randomly to cubicles, after which the experimenter read the instructions aloud. A printed copy of the instructions including the payoff table was also dealt to each cubicle. All choices were made privately and anonymously on the computer. Each session started with two practice rounds that did not influence participants’ earnings from the experiment.

**Fig. 1:**
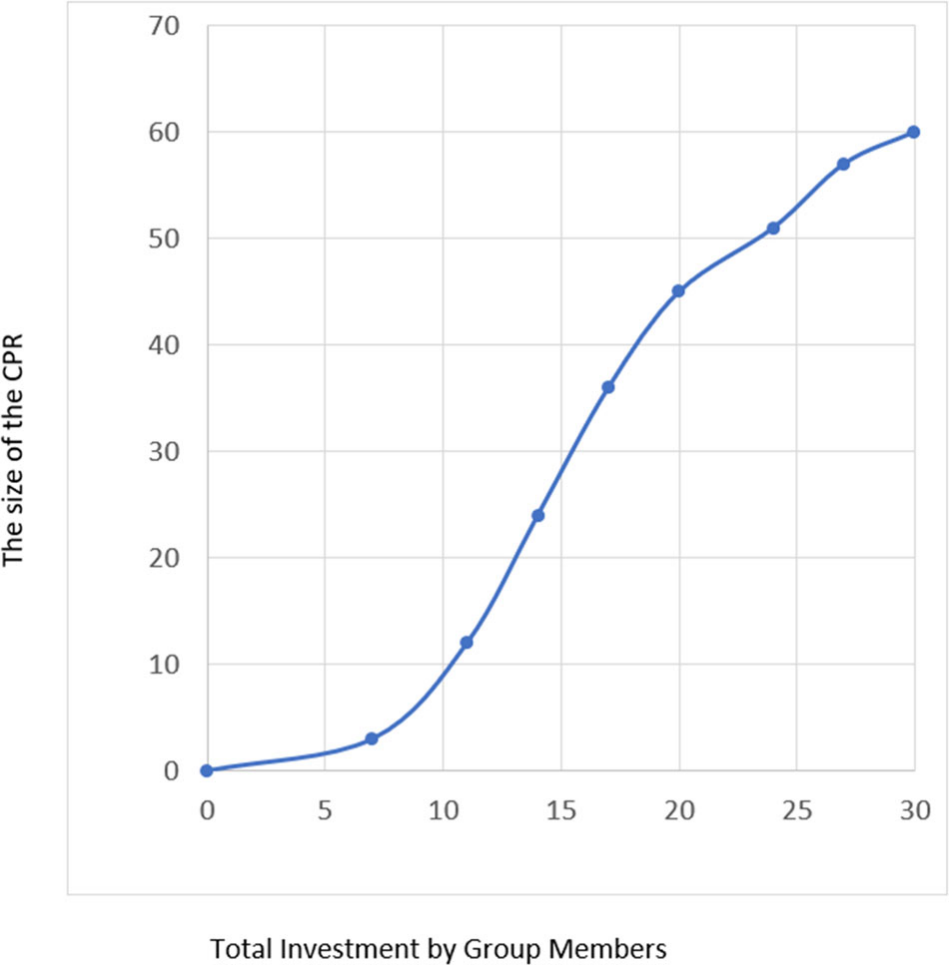
The common pool resource as a function of total contributions

In the experiment, participants played the game in groups of three (formed randomly). Each participant was granted 10 points at the beginning of each round (1 point = 50 cents). Points could either be contributed to the common pool resource infrastructure or kept to oneself. Points contributed to the common pool resource infrastructure determined the size of the resource. Each group played the game for ten rounds, and the participants were aware of the number of rounds before they started. The groups remained the same throughout the rounds. Each participant made two decisions per round: (1) How much to contribute to the resource infrastructure, and (2) how much to extract from the resource. Table 1 represents the payoff table that was shown to participants. The size of the common pool resource as a function of the total contributions is also depicted in Fig. 1.

**Table 1:** The payoff table

Total points contributed to the common pool resource infrastructure by the group members	Size of the common pool resource in points
0–6	0
7–10	3
11–13	12
14–16	24
17–19	36
20–23	45
24–26	51
27–29	57
30	60

The common pool growth function resembles loosely a sigmoid function, which in this context means that up until 19 units contributed, the growth of the resource exhibits increasing returns to scale, after which the extra units contributed begin to yield smaller marginal increases in the size of the resource. The payoff table is specified so that the Nash equilibrium is to invest nothing, and the Pareto-optimal outcome ensues if each group member contributes all of her or his initial endowment to the common pool.

All points a participant did not contribute, and all points he or she extracted, were added to the participant’s earnings from the experiment. Contributions to the common pool resource infrastructure were made simultaneously but extractions were made in a pre-determined random order of the three group members. The order remained fixed throughout the ten rounds the game was played. Maximum amount of extraction at any position was set to 30 points. This means that the players in the first two positions could exhaust the whole resource even if the group had managed to produce the maximum amount.

**Fig. 2:**
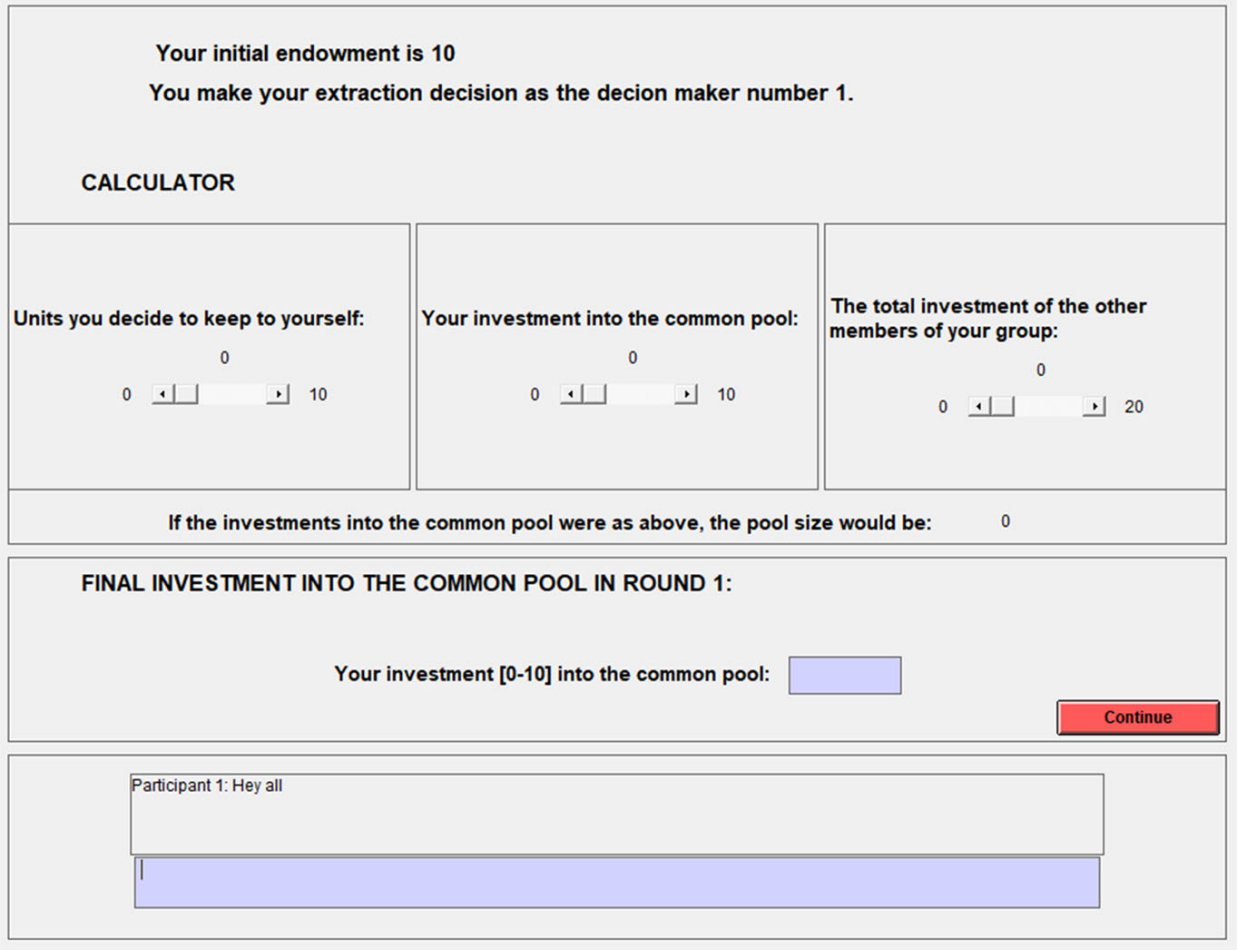
The screen for making contribution decisions, with the chat box

The endowments and the common pool resource were reset at the beginning of each round, that is, a new round started with all group members having 10 points each and zero contributions to the common resource. Figure 2 shows an English translation of the contribution screen from the *Communication* and *Deliberation* treatment conditions. The three frames in the middle allow the participant to experiment with different sums. The lowest box, available only in the *Communication* and *Deliberation*, allows to write chat entries. Apart from the chat box the screen was identical in *Baseline*.

After 10 rounds were completed, subjects filled in a questionnaire that did not influence their earnings. The questionnaire included items on academic success, social and political trust, 13 items on political attitudes, background variables, and an open question asking participants to characterize the chat discussions in their group. To determine participants’ earnings from the experiment, one round was selected at random for each participant and points received from that round changed into euros. Participants were paid a show-up fee of five euros plus their earnings from the randomly selected round. Thereafter they could leave the experiment. The experiment lasted about 1–1.5 h for each participant.

## Results

The experimental sessions were conducted between November 2019 and March 2020 at the PCRClab, University of Turku. The participants (*n* = 168) were recruited through the laboratory’s subject pool, and they were mainly undergraduate and graduate students, representing various fields of study (mean age = 28.5, 68% female, and 7% economics or business students). Table 2 shows the assignment of participants into the three treatment conditions and into groups of three.

**Table 2:** The treatment groups

	*Baseline*	*Communication*	*Deliberation*
Subjects	*n* = 54	*n* = 51	*n* = 63
Groups	*n* = 18	*n* = 17	*n* = 21

### Contributions to the Common Pool Resource Infrastructure

We start by examining contributions to the infrastructure. All the analyses in this section are performed with a group as the basic unit of observation. Statistical significance is Bonferroni corrected to address multiple comparison where appropriate. Table 3 presents means and standard deviations for each treatment condition and for each player position aggregated over all ten rounds. The table shows that the amount of points contributed to the common pool resource infrastructure is different from zero in each treatment condition. In *Baseline*, the observed *t*-statistics was *t*(17) = 13.24, *p* = 2.211e^−10, in *Communication t*(16) = 18.74, *p* = 0.2.61e^−12, and in *Deliberation* *t*(20) = 32.44, *p* = 2.2e^−16.

**Table 3:** Mean contributions to the common pool resource infrastructure by treatment conditions and player positions (standard deviations in parenthesis)

	*Baseline*	*Communication*	*Deliberation*
Over all positions	19.84 (8.12)	25.62 (7.02)	27.59 (5.76)
1^st^ position	7.97 (2.06)	9.09 (1.47)	9.56 (0.84)
2^nd^ position	6.54 (2.80)	8.50 (2.16)	9.00 (2.00)
3^rd^ position	5.33 (2.87)	8.03 (2.62)	9.03 (1.62)

On the aggregate level, the total amounts contributed to the common pool resource infrastructure were greater in *Communication* than in *Baseline* (One-tailed Mann–Whitney *U*: *p* = 0.005). Likewise, *Deliberation* induced a considerably higher average contribution rates compared to *Baseline* (One-tailed Mann–Whitney *U*, *p* = 0.00002). The difference between *Communication* and *Deliberation* is not statistically significant at the conventional level (One-tailed Mann–Whitney *U*: *p* = 0.0815). This implies that H1 (*an opportunity to communicate increases contributions to the asymmetric common pool resource infrastructure*) is supported because both modes of communication, *Communication* and *Deliberation*, induced more contributions than *Baseline*. The difference between *Communication* and *Deliberation* is not statistically significant, indicating that H2 (*structured communication increases contributions to the asymmetric common pool resource infrastructure more than unstructured communication*) does not get support.

In addition to total means, Table 3 represents mean contributions in each player position. The mean values suggest that moving from *Baseline* to *Communication*, and from *Communication* to *Deliberation* attenuates differences between player positions. To test H3 (*without communication, contributions to the asymmetric common pool resource infrastructure decrease as a function of player position)*, we analyse first differences in contributions between the player positions in *Baseline* with Matched-Pairs Signed Ranks test, one tailed. The analysis reveals that the differences in contributions between the first and second movers (*U*(17) = 75, *p* = 0.0186), as well as the first and third movers are statistically significant (*U*(17), *p* = 0.00075), whereas the difference between the second and third movers just fails to be statistically significant (*U*(17), *p* = 0.0504). In C*ommunication* (*U*(16), *p* = 0.015) and *Deliberation* (U(20), *p* = 0.0285), the difference between the first and the third mover is statistically significant, whereas other differences are not. After correcting for multiple comparisons, the differences between the first and third movers remain statistically significant both in *Baseline* and in *Communication*, whereas in *Deliberation*, the difference between the first and the third mover is no longer significant. This suggests that regarding the influence of players’ positions on their contribution decisions, *Baseline* and *Communication* were different from *Deliberation*. Engaging in deliberation attenuated the influence of participants’ positions, whereas engaging in communication did not, giving support to H4 (*structured communication attenuates the position effect on contributions to the asymmetric common pool resource infrastructure more than unstructured communication*).

**Fig. 3:**
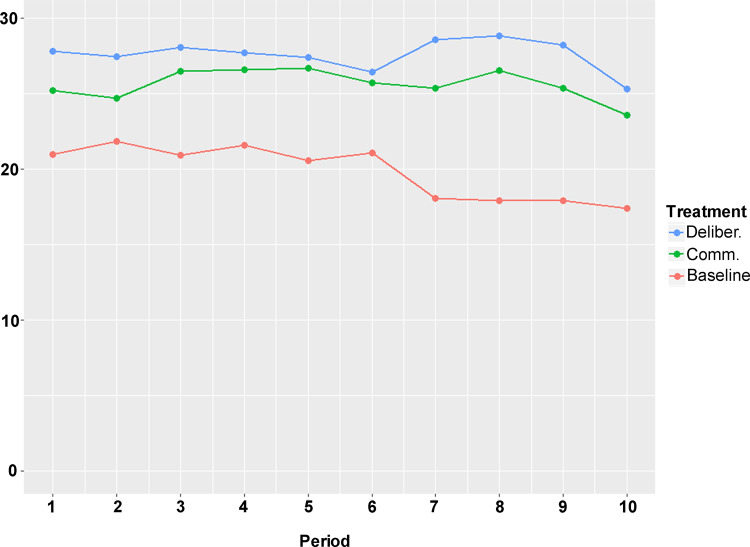
Groupwise mean contributions over 10 rounds

To test H4 further, we turn to regression analysis to take into account the dynamic nature of the experiment (10 periods, matched groups). Figure 3 visualizes the group mean contribution over the 10 periods in each treatment condition. The figure reveals an end-game effect because average contributions drop towards the end of the ten rounds. Moreover, *Communication* and *Deliberation* give rise to bigger average contributions to the common pool resource infrastructure in comparison to *Baseline* throughout the rounds, *Deliberation* also being slightly above *Communication*.

The dependent variable in the regression model is the sum of participants’ contributions to the common pool resource infrastructure (Table 4). Player position, treatment and their interaction are independent variables. The regression results show that the player position had a strong negative effect on contributions. This is reflected in the large and highly significant regression coefficient (position = −1.32, *p* = 0.0012). In *Deliberation*, player position had a significant effect on contributions such that players in later positions contributed more to the common pool resource infrastructure compared to what players in later positions did in conditions without deliberation. This is seen in the highly significant and relatively large positive interaction term (*Deliberation* × Position = 1.055, *p* = 0.0191). Such interaction is not observed between *Communication* and position. The regression analysis gives support to the initial observation that *Deliberation* increased player contributions in positions 2 and 3, and in so doing evened out the differences in contributions between player positions. H4 is thereby supported.^3^

**Table 4:** Regression analysis of contributions to the common pool resource infrastructure, robust standard errors clustered on group and time (period)

	Estimate	Std. Error	*T*-val.	Pr(>|t|)
Constant	9.252	0.812	11.397	<0.000001
Communication	0.354	1.024	0.346	0.73
Deliberation	0.474	0.876	0.541	0.589
Position	−1.319	0.405	−3.255	0.001**
Communication × Position	0.787	0.538	1.463	0.144
Deliberation × Position	1.055	0.45	2.346	0.019*

*Adjusted R^2: 0.159. Balanced panel, *n* = 168, *T* = 10, *N* = 1680

Significance levels: ***p < 0.001, **p < 0.01, *p < 0.05

### Extractions from the Common Pool Resource

We have now seen that participants succeeded in providing infrastructure for an asymmetric common pool resource. How did they then use the resource, and did the treatment and player positions influence resource use? Table 5 reports the total common pool resource, averaged over all 10 rounds in each treatment condition, and the respective shares of the theoretical maximum size of the resource (60). Due to a nonlinear production function, smaller total contribution levels lead to considerably lower average of total common pool resource allocation in *Baseline*, compared to the two other conditions. Table 5 shows that *Communication* (84%) and even more *Deliberation* (92%) come rather close to the theoretical maximum.

**Table 5:** Groupwise mean sizes of the common pool resource and proportion of the theoretical maximum size

Treatment	mean	s.d.	Share
*Baseline*	37.35	20.19	62.30%
*Communication*	50.24	16.84	83.70%
*Deliberation*	54.94	13.11	91.60%

**Fig. 4:**
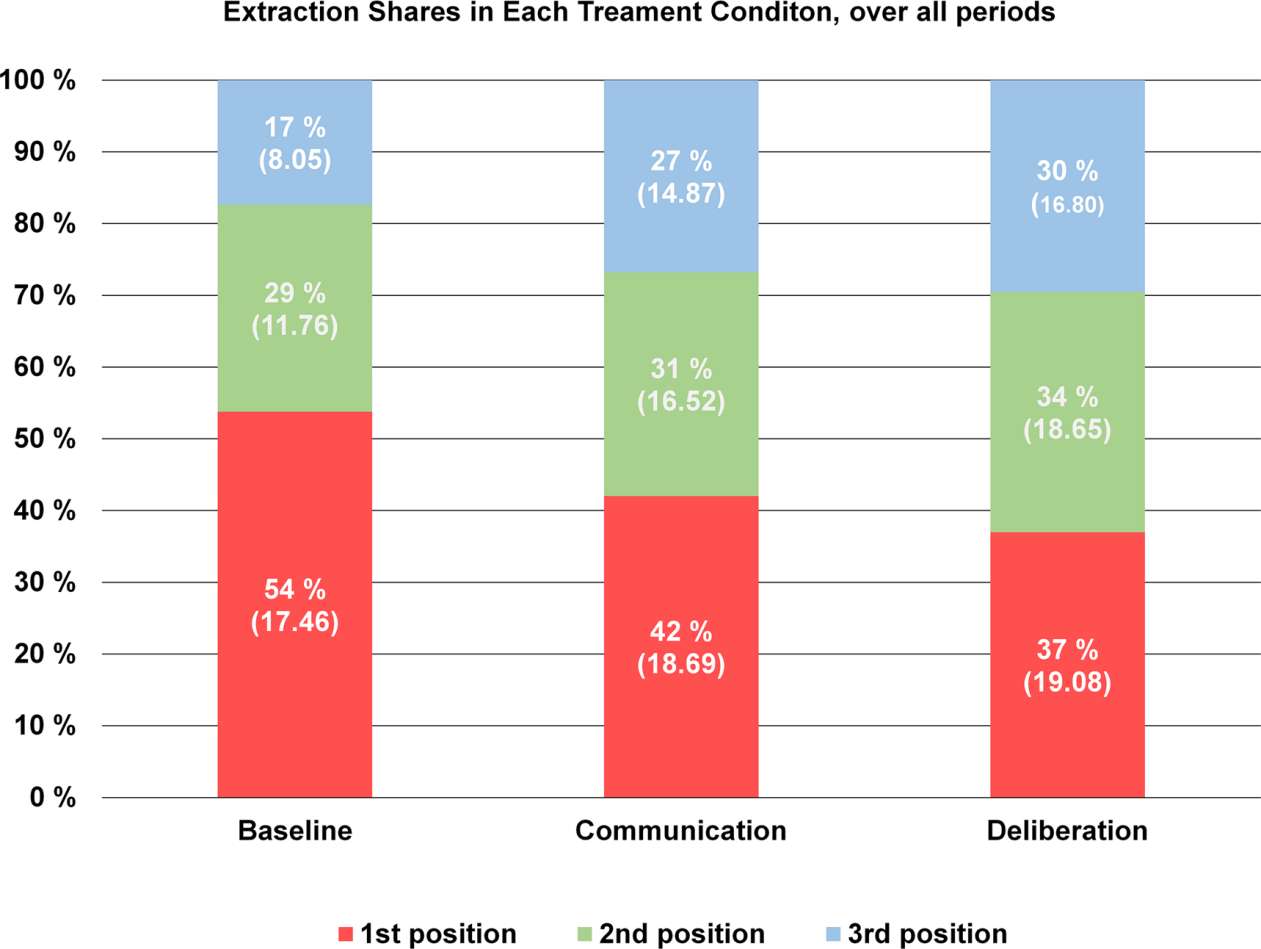
Shares extracted in each player position, per treatment condition. Averages in terms of absolute points in parenthesis

Figure 4 displays the *relative* shares of extractions in each player position per treatment. In *Baseline*, the first movers extracted significantly larger relative shares of the common pool resource than in either treatment condition (*Baseline* vs. *Communication* Mann–Whitney *U*, *p* = 0.011; *Baseline* vs. *Deliberation* *p* = 0.00009). However, the absolute size of the total resource, on average, was smaller in *Baseline*, so in terms of number of points extracted, the first movers’ share was not any larger in *Baseline* than in *Communication* or *Deliberation* (cf. the mean extraction size in absolute terms in parenthesis). In *Baseline*, the second movers extracted a relatively larger share of the remaining resource compared to either *Communication* or *Deliberation* (*Baseline* vs. *Communication* Mann–Whitney *U*, *p* = 0.021; and *Baseline* vs. *Deliberation* *p* = 0.006, respectively), which left a considerably smaller share for the 3rd mover, on average. The difference between *Communication* and *Deliberation* with respect to shares extracted is not statistically significant.

To elaborate further on the distributions of extractions, we calculated Gini coefficients for each treatment condition. The Gini coefficient varies between 0 (complete equality, indicating equal shares for each member of the group) and 1 (complete inequality, a single individual commanding the whole resource). We utilized the R Package DescTools for computing the Gini coefficients (Signorell et al., (2020)). The mean of points extracted for each individual position in each group over all the rounds were first computed (also listed in Table S1, Supplementary material). We then used the mean values to calculate a Gini coefficient for each group. Table 6 displays the grand average for each treatment condition, taken over the groupwise mean Gini coefficients.

**Table 6:** Average groupwise Gini coefficients in each treatment condition

Treatment	Average Gini coefficient	s.d
*Baseline*	0.34	0.23
*Communication*	0.12	0.20
*Deliberation*	0.06	0.08

Table 6 shows that the average Gini coefficient value is significantly lower in *Communication* than in *Baseline* (*t*(32.73) = 2.96, *p* = 0.017, two sided *t*-test, equality of variances not assumed). Likewise, the difference between the average Gini coefficient in *Baseline* and in *Deliberation* is statistically significant (*t*(20.072) = 4.85, *p* = 0.00029), whereas the difference between *Communication* and *Deliberation* is not (*t*(19.69) = 1.19, *p* = 0.74). The analysis of the Gini coefficients gives further support to our results obtained through comparing average shares extracted in each player position, that is, both treatment conditions are statistically different from *Baseline*, but the difference between *Communication* and *Deliberation* is not statistically significant.

We can summarize the overall results regarding common pool resource extractions as follows: On average, *Communication* and *Deliberation* generated a larger total common pool resource in terms of points contributed, and they also induced a more equal distribution in terms of units extracted. We thereby conclude that H5 (*structured communication leads to a more equal distribution of extractions from the asymmetric common pool resource compared to unstructured communication or no communication*) is only partly supported, because although a difference between *Baseline* and the other two conditions was observed, *Communication* and *Deliberation* did not produce significantly different extraction distributions.

### Analysis of Chat Entries

We did not formulate hypotheses on chat entries but rather ask: Is there a difference between *Communication* and *Deliberation* regarding the types of discussions participants had in the chat? Moreover, do players’ positions influence their chat behaviour, and if they do, is the influence contingent on the treatment? Analysis of the length of discussions (number of words) reveals that *Deliberation* induced clearly longer discussions compared to *Communication*. The total number of words used in *Deliberation* was 915, whereas in *Communication* it was 369. The corresponding mean values are 4.29 and 2.96 (F = 31.83, *p* = 0.000). The player position also had an effect, both in *Deliberation* and in *Communication*, the first and third movers spoke more than the second movers (F = 3.82, *p* = 0.022). There were no differences between the treatment conditions, that is, the effect of player position on chat length was not conditional on treatment (F = 0.89, *p* = 0.413).^4^

We also classified chat entries according to the type of content.^5^ The analysis of chat contents revealed that the rules applied in *Deliberation* influenced participants’ tendency to justify their propositions. A larger share of propositions contained a justification in *Deliberation* (24 %) compared to *Communication* (6 %) (χ^2^ = 15.26, *p* = 0.000). Player position did not influence the use of justifications in either condition. A counterintuitive observation was that normative principles were somewhat more often mentioned in *Communication* than in *Deliberation* (4.3% vs. 1.9%; χ^2^ = 23.426, *p* = 0.005). This is curious because one would think that justifications would include normative principles and they would thereby be more often presented in *Deliberation*. Participants did not make many comments about other group members (less than 10% of chat entries were comments about others), but the treatment influenced commenting (χ^2^ = 9.236, *p* = 0.010). Interestingly, negative comments about others were more common in *Deliberation* than in *Communication* (4.9% vs. 1.4%; χ^2^ = 14.506, *p* = 0.006), whereas there was not so much difference between the treatment conditions regarding positive comments (3.2% vs. 4.6%).

## Discussion and Conclusion

We studied the influence of communication and deliberation on contribution and extraction decisions and the influence of player position in an asymmetric common pool resource game. Deliberation was guided by specific rules that encouraged justifications, perspective-taking and respect. We observed that more contributions to the common pool resource infrastructure were made under both modes of communication compared to baseline; that the mode of communication did not influence total contributions or extractions; and that player positions had an effect on contributions, but that unlike communication deliberation attenuated the influence of power asymmetries.

The discussion rules applied in *Deliberation* may have influenced the position effect either by a direct effect on behaviour or by an indirect effect via an influence on the kinds of discussions participants engaged in. Differences in the chat entries in *Deliberation* and *Communication* suggest that the influence went at least partly via the chat. In *Deliberation*, participants both talked more, and tended to provide justifications for their views. This may have contributed to increased trust between players in different positions. Simply talking more may increase trust, but justifications can also be an important element in building trust because fair actions are easier to justify. We suggest that the rules of discussion have influenced participants via promoting discussions that build trust between the players, which in turn have equalized expectations about how the game will be played in different player positions.

A somewhat counterintuitive observation was that normative principles were more often mentioned in *Communication* than in *Deliberation*. We are not able to explain this observation, but one could speculate whether the instruction to consider others’ perspectives in *Deliberation* derived attention to individuals’ positions rather than to general principles. Another unexpected observation was that negative comments about others were more common in *Deliberation* than in *Communication*. Negative comments were mainly about other participants who were not playing according to what was agreed on in the group and it was the third movers who mainly made these types of comments in *Deliberation*. It is possible that in *Deliberation*, third movers were concerned about fairness and did not accept a “first mover advantage”, that is, the first mover’s right to take advantage of her or his position. There is evidence that participants make different demands depending on their position in sequential games (Budescu and Au 2002), suggesting that later movers may often accept their inferior position and adapt their own requests accordingly. In *Deliberation*, the third movers may have instead thought that everyone has an equal right to the benefits of the common resource. So, when someone did not follow what was agreed on, the third movers were inclined to comment on this. If this was the case, rules of discussion would have enforced a kind of monitoring and verbal sanctioning mechanism.

It is noteworthy that while the discussion rules we applied in *Deliberation* fall short of genuine deliberation, our observations are somewhat similar to a large evidence form deliberative mini-publics. Many mini-public studies show evidence of preference transformations (Barabas, 2004, Hansen and Andersen 2004; Fishkin 2009; Grönlund et al. 2015), as well as increases in social trust (Grönlund et al. 2010), perspective-taking (Grönlund et al. 2017; Muradova 2020), and readiness for collective action (Grönlund et al. 2010). All of these processes can have a role in contributions to social dilemmas.

Certain limitations pertain to our research. First, it is possible that the manipulation we used, that is, the rules of discussion in *Deliberation*, was not strong enough to produce a difference in contributions between the treatment conditions. For example, combining rules to face-to-face discussions may have produced a larger difference. Some studies support the effectiveness of face-to-face discussion (Baillet 2010), but others do not (Bochet et al. 2006). Furthermore, a ceiling effect may influence the non-significant finding between *Communication* and *Deliberation*. Participants contributed substantial amounts to the common pool resource infrastructure, and both *Communication* and *Deliberation* enabled contributions that were close to the theoretical maximum (84 and 92 precents respectively), whereas in *Baseline* contributions were clearly lower (62 percent of the maximum). *Communication* also produced a rather even distribution of extractions indicating that *Deliberation* could not do much better. When the potential difference between *Communication* and *Deliberation* is not extensive, our number of participants may have been too small to detect a statistically significant difference. Moreover, it is possible that the wording of the instructions influenced the level of contributions (Ramalingam et al. 2018). Unlike Janssen et al. (2011a; 2011b; 2015), we did not frame the choice task in terms of a real world common pool resource problem, but rather talked about a “common pool resource”. However, it is notable that with a similar experimental set up Janssen et al. (2011a; 2011b) also observed substantial amounts of contributions. More importantly, the same terminology was used in each treatment condition, and the *Baseline* gave rise to less contributions compared to *Communication* and *Deliberation*. Finally, one could ask whether the discussion rules applied in *Deliberation* primed social desirability in a manner *Baseline* or *Communication* did not. We cannot rule out this possibility, but we also like to point out that the same would apply to similar rules if used in the field implying that social desirability should not undermine the generalizability of our results.

Our study gives preliminary support for the view that deliberation helps to overcome power imbalances and suggests that deliberation can produce sustainable outcomes in situations where common pool resources are especially hard to manage. This is a promising result because applying similar rules in the field to solve (local) common pool resource problems would be a cost-effective method to promote sustainable common pool resources use. We acknowledge though that more research, in particular field experiments, is needed to elaborate further on the potential of deliberation to help sustainable resource use and neutralizing power imbalances.

## Supplementary Information

Supplementary material

## Footnotes

1 It is noteworthy that we use the ideals of deliberative democracy to derive rules of communication, but otherwise we do not apply the central tenets of deliberation, such as a representative pool of participants or moderated discussions.

2 See Supplementary material for the experimental instructions.

3 Table S1 in the Supplementary material provides panel regression results with gender and economics / business major controlled. The background variables did not have a statistically significant effect on contributions to the common pool resource infrastructure.

4 Table S3, Supplementary material, represents chat lengths in words per treatment condition and player position.

5 The contents of the chats were coded according to six criteria: type of comment, type of question, type of answer, justification, content of entry and comments about others. Two graduate students who had not participated in the research project coded the chat entries. Variables Treatment and Player position were removed from the data file before coding. Comparing the two coders reveals a satisfactory degree of agreement measured with Cohen’s κ. Type of entry: κ = 0.314, *p* = 0.000; Type of question: κ = 0.451, *p* = 0.000; Type of answer κ = 0.401, *p* = 0.000; Justification κ = 0.308, *p* = 0.000; Content of entry κ = 0.306, *p* = 0.000; Comments about others κ = 0.359, *p* = 0.000.
