# Supplementary material for: The Mode of Communication as a Driver of Sustainable and Equitable Asymmetric Common Pool Resource Use
Source: Environ Manage. 2023 Apr 28;72(1):190–202. doi: 10.1007/s00267-023-01825-w (PMC10220137; doi:10.1007/s00267-023-01825-w)
Supplement: Supplementary file 1 — Supplementary material [file 267_2023_1825_MOESM1_ESM.docx]

**Supplementary material**

**Table S1. Panel estimation with background variables***

|  | **Estimate** | **Std. Error** | **t value** | **Pr(>\|t\|)** |
| --- | --- | --- | --- | --- |
| (Intercept) | 9.09 | 0.860 | 10.57 | < 0.000001*** |
| Communication | 0.52 | 1.1007 | 0.472 | 0.64 |
| Deliberation | 0.59 | 0.902 | 0.655 | 0.51 |
| Position | -1.39 | 0.417 | -3.32 | <0.001*** |
| Gender | 0.12 | 0.379 | 0.322 | 0.75 |
| Econ/Business major | 0.52 | 0.618 | 0.848 | 0.40 |
| Communication x Position | 0.78 | 0.571 | 1.36 | 0.175 |
| Deliberation x Position | 1.08 | 0.466 | 2.31 | 0.0201* |
| Significance levels: *** p < 0.001, ** p < 0.01, * p < 0.05, . p < 0.10. | | |  |  |
| Adj. R^2: 0.17, Balanced Panel, n=152, T=10, N=1520 |  |  |  |  |

*Table S2. reports the result of the panel estimation with relevant background variables controlled. We detect no gender effect. Moreover, business/economics major students’ contributions towards the common pool resource infrastructure seem to be on the level of the rest of the subjects.

**Table S2. Groupwise mean extraction and Gini indices**

| Group ID | Extraction 1 | Extraction 2 | Extraction 3 | Treatment | Gini Index |
| --- | --- | --- | --- | --- | --- |
| 21 | 19.50 | 18.10 | 19.40 | 0 | 0.02 |
| 22 | 22.30 | 16.60 | 18.10 | 0 | 0.10 |
| 23 | 20.10 | 8.70 | 8.70 | 0 | 0.30 |
| 24 | 20.00 | 12.20 | 10.40 | 0 | 0.23 |
| 25 | 15.50 | 17.00 | 12.50 | 0 | 0.10 |
| 26 | 11.70 | 9.70 | 1.40 | 0 | 0.45 |
| 41 | 6.00 | 2.10 | 0.00 | 0 | 0.74 |
| 42 | 19.70 | 18.40 | 17.40 | 0 | 0.04 |
| 43 | 22.20 | 4.60 | 3.20 | 0 | 0.63 |
| 44 | 15.70 | 10.40 | 0.00 | 0 | 0.60 |
| 45 | 17.80 | 19.20 | 12.80 | 0 | 0.13 |
| 46 | 22.60 | 14.20 | 0.70 | 0 | 0.58 |
| 51 | 14.60 | 10.20 | 5.80 | 0 | 0.29 |
| 52 | 15.20 | 10.80 | 8.80 | 0 | 0.18 |
| 53 | 20.70 | 9.80 | 3.70 | 0 | 0.50 |
| 54 | 23.10 | 8.50 | 8.00 | 0 | 0.38 |
| 55 | 4.60 | 1.70 | 0.30 | 0 | 0.65 |
| 56 | 22.00 | 19.50 | 13.70 | 0 | 0.15 |
| 71 | 11.90 | 9.30 | 5.20 | 1 | 0.25 |
| 72 | 19.00 | 20.00 | 11.30 | 1 | 0.17 |
| 73 | 18.70 | 18.70 | 18.70 | 1 | 0.00 |
| 74 | 19.60 | 19.00 | 14.80 | 1 | 0.09 |
| 75 | 20.50 | 20.00 | 18.00 | 1 | 0.04 |
| 81 | 20.00 | 20.00 | 20.00 | 1 | 0.00 |
| 82 | 20.00 | 20.00 | 20.00 | 1 | 0.00 |
| 83 | 12.20 | 11.50 | 9.30 | 1 | 0.09 |
| 84 | 18.80 | 18.90 | 18.70 | 1 | 0.00 |
| 85 | 20.00 | 20.00 | 20.00 | 1 | 0.00 |
| 86 | 20.30 | 16.90 | 16.50 | 1 | 0.07 |
| 91 | 20.00 | 20.00 | 20.00 | 1 | 0.00 |
| 92 | 12.40 | 3.90 | 5.00 | 1 | 0.40 |
| 93 | 23.20 | 16.80 | 15.80 | 1 | 0.13 |
| 94 | 21.70 | 6.30 | 0.00 | 1 | 0.78 |
| 95 | 19.50 | 19.50 | 19.50 | 1 | 0.00 |
| 96 | 20.00 | 20.00 | 20.00 | 1 | 0.00 |
| 11 | 20.00 | 20.00 | 20.00 | 2 | 0.00 |
| 12 | 20.00 | 20.00 | 20.00 | 2 | 0.00 |
| 13 | 20.50 | 19.50 | 19.10 | 2 | 0.02 |
| 14 | 20.00 | 20.00 | 20.00 | 2 | 0.00 |
| 15 | 20.00 | 20.00 | 20.00 | 2 | 0.00 |
| 16 | 21.00 | 19.50 | 18.00 | 2 | 0.05 |
| 31 | 18.50 | 18.10 | 8.50 | 2 | 0.22 |
| 32 | 12.50 | 11.00 | 6.40 | 2 | 0.20 |
| 33 | 20.00 | 20.00 | 20.00 | 2 | 0.00 |
| 34 | 20.20 | 19.80 | 18.80 | 2 | 0.02 |
| 35 | 20.00 | 20.00 | 20.00 | 2 | 0.00 |
| 61 | 19.70 | 19.70 | 19.70 | 2 | 0.00 |
| 63 | 16.00 | 13.00 | 10.30 | 2 | 0.15 |
| 64 | 19.10 | 20.00 | 12.20 | 2 | 0.15 |
| 65 | 20.00 | 20.00 | 20.00 | 2 | 0.00 |
| 101 | 15.50 | 9.70 | 11.10 | 2 | 0.16 |
| 102 | 20.00 | 20.00 | 20.00 | 2 | 0.00 |
| 103 | 20.00 | 22.00 | 18.00 | 2 | 0.07 |
| 104 | 20.00 | 20.00 | 20.00 | 2 | 0.00 |
| 105 | 18.00 | 19.00 | 17.00 | 2 | 0.04 |
| 106 | 19.70 | 20.40 | 13.60 | 2 | 0.13 |

**Table S3. Length of chat entry in words by treatment and position**

| Treatment | Position | Words mean (SD) | | N |
| --- | --- | --- | --- | --- |
| Deliberation | 1 | 4.83 | (4.42) | 292 |
|  | 2 | 3.72 | (3.80) | 302 |
|  | 3 | 4.34 | (4.04) | 321 |
| Total |  |  |  | 915 |
| Communication | 1 | 3.04 | (2.89) | 91 |
|  | 2 | 2.67 | (2.30) | 141 |
|  | 3 | 3.22 | (2.90) | 137 |
| Total |  |  |  | 369 |

**Experimental instructions [DELIBERATION TREATMENT]**

How to interpret the instructions:

Instructions in plain text were available in all three treatments.

**Instructions in bold text: Added to Baseline instructions in both Communication and Deliberation treatments**

**Instructions in bold and red: Added to Deliberation treatment instructions**

All the instructions were in plain text in each treatment in the actual experiment. Red color and bold style were added to the Supplementary Material to highlight the differences between instructions in the respective treatments.

**Instructions**

Welcome to the decision-making experiment!

Please do not talk during the experiment. If you have any questions, please raise your hand, so the instructor will help you personally. Please turn off your mobile phone.

Each participant will receive a show up fee of 5 euros. In addition, you can earn more money from the experiment.

The rewards will be paid in private right after the experiment is over, either via mobile transfer or in cash. When you are ready, please wait at your seat until you are asked to move to the next room for your payment.

You will make your decisions privately and anonymously through computer interface. All the results will be analyzed statistically, and the experimenters will be not able to identify individual participants by their choices.

You will be member of a three-person group, which is randomly formed. Each group member is given an order number 1, 2, or 3 that matches their moving order. Moving order is randomly determined. You will remain within the same group throughout the whole experiment, and your order number will also remain the same. The decisions you make will affect only your own earnings and the earnings of the two other members in your group. You will not get to know the names or other personal details of other the members in your group.

Your task in the experiment is to invest experimental currency into a common pool and extract experimental currency from this common pool. The experiment comprises of 10 rounds, and your initial endowment in each round is 10 units of the experimental currency. At the end of the experiment, experimental currency is converted to EUR, and the exchange rate is: 1 currency unit = 50 cents.

In each round you will make two decisions. First you will decide how much of your initial endowment you will invest into the common pool, and how much you will keep to yourself. The second decision determines how much you will extract from the common pool. The money you extract from the common pool will be added to the money you kept to yourself of the initial endowment. The maximum amount you can extract per round is 30 currency units, provided that the common pool has at least that many units when it is your turn to extract.

Available resources are not transferred between the rounds. That is, in the beginning of each round, initial endowment and the common pool are ‘reset’. In particular, if all the units are not extracted from the common pool, these are not transferred to the following round. Likewise, the initial endowment is 10 units in each round irrespective of how much you invested to the common pool in the previous round.

The members of your group make their investment decisions simultaneously, but extraction decisions are made in a sequential order dictated by the order numbers.

**In each round, you have an opportunity to use a chat box on your screen to discuss with other members of your group before everyone makes their investment and extraction decisions privately. The chat box displays the order number of each discussant but no other details. You have a chance to discuss in each round before decision making. Time reserved for discussion is one minute. Please do announce to other discussants when you exit the chat.**

**Each member of your group sees what you type in the chat box, and you see the typing of every other member in your group, respectively.**

**You can freely discuss how to act as a group, but the discussion is subject to the following rules: You need to justify your investment and extraction decisions. You also need to try to understand the point of view of the other members of your group. The chat messages cannot contain details that could be used to identify any person in your group (e.g. names, physical characteristics), and the messages cannot be threatening or derogatory.**

Your reward from the experiment is determined by how much you have kept from the initial endowment to yourself and how much you have extracted from the common pool.

More detailed instructions about investing and extracting are displayed on the computer screen. You also have available throughout the experiment the table in the next page, which shows you how the investments generate the common pool.

The experiment begins with two practice rounds that do not affect your rewards. The purpose of these tasks is to rehearse decision making in the experiment.

After the practice rounds are over, the actual experiment will begin, and it will determine your rewards from the experiment. The beginning of the actual experiment will be announced clearly.

After the experiment is over, you are expected to fill a questionnaire. This won’t affect your reward. The URL of the questionnaire is written on a paper sheet that is dealt onto your desk. Please type it in a web browser so you can fill in the questionnaire.

Does anyone have any questions at this stage?

The table below shows how the common pool resource grows as the group members invest experimental currency. For instance, if the summed-up total of all three investments is 3 currency units, the common pool size is 0. If the total investments are 18, the common pool size is 36. If the total investment is 27, the common pool size is 57, etc.

| **The total of the investments by the group members into the common pool** | **The Common Pool Size** |
| --- | --- |
| **0-6** | **0** |
| **7-10** | **3** |
| **11-13** | **12** |
| **14-16** | **24** |
| **17-19** | **36** |
| **20-23** | **45** |
| **24-26** | **51** |
| **27-29** | **57** |
| **30-30** | **60** |
